# Lane line detection based on cross-convolutional hybrid attention mechanism

**DOI:** 10.1038/s41598-025-01167-z

**Published:** 2025-05-09

**Authors:** Jianping Wen, Zhuang Zhao, Chenze Wang, Ze Sun, Chao Xu

**Affiliations:** College of Mechanical Engineering, Xi’an University of Science and Technology, Xi’an, 710054 China

**Keywords:** Autonomous driving, Lane line detection, Cross-Convolution, Attention mechanism, Neural networks, Mechanical engineering, Computer science

## Abstract

In order to enhance the accuracy and robustness of lane line recognition in dynamic and complex environments, this paper proposes a lane line detection model based on a cross-convolutional hybrid attention mechanism (CCHA-Net). Unlike traditional approaches that separately employ channel and spatial attention, our proposed mechanism integrates these modalities through cross-convolution, thereby enabling cross-group feature interaction and dynamic spatial weight allocation. This novel integration not only improves the continuity of elongated lane features but also enhances the model’s ability to capture long-range dependencies in challenging scenarios. Additionally, this paper designs a lightweight message-passing module that employs dual-branch multi-scale convolutions to achieve cross-spatial domain feature fusion while reducing the number of parameters. Experimental results demonstrate that CCHA-Net achieves an F1 score of 80.2% on the CULane dataset and an accuracy of 96.8% on the TuSimple dataset, effectively enhancing lane line recognition accuracy in ever-changing and intricate environments.

## Introduction

Lane line detection is a fundamental component of autonomous driving systems and plays a critical role in vehicle navigation and safety. As the field of autonomous driving continues to evolve, significant research efforts have been dedicated to improving lane detection techniques due to its pivotal importance. In real-world operations, autonomous vehicles face a variety of challenging road conditions. These include occlusions by other vehicles or roadside objects, drastic lighting variations due to shadows, glare, and weather-induced changes, as well as other environmental factors that introduce uncertainties and variability into the driving scene^1–4^. Such complexities can severely affect the performance and reliability of lane detection systems.

Several key features directly impact the accuracy of lane detection. For instance, the distinctiveness of lane edges is crucial, as clear boundaries facilitate accurate localization of the lane^5,6^. Color information is another significant feature, since lane markings often have specific color characteristics that help in distinguishing them from the road surface^7^. Additionally, the geometric properties of lane markings, including curvature and width, provide essential context that influences the detection algorithms^8^. Effective extraction and integration of these features are critical for developing robust lane detection systems.

Traditionally, lane detection methods have relied on classical image processing techniques. Edge detection algorithms, such as the Sobel operator^9^ and the Canny edge detector^10^, are commonly used to extract the contours of lane lines. Following edge detection, the Hough Transform^11,12^ is typically employed to identify and parameterize the linear structures corresponding to lane lines in an image. These methods offer simplicity and computational efficiency, making them attractive for early lane detection applications.

To enhance detection precision, a number of preprocessing techniques have been introduced. For example, Gaussian filtering^13^ is used to reduce image noise, which can otherwise lead to false edge detection and poor segmentation. The inverse perspective transformation^14^ is applied to convert the perspective view of the road into a bird’s-eye view, thereby simplifying the geometry of the lanes and making subsequent detection steps more robust. Furthermore, various image enhancement techniques^15^ are used to improve the visibility of lane markings under different illumination conditions.

Despite these advancements, traditional lane detection algorithms are limited by their reliance on manually engineered features. This dependency restricts their ability to generalize to diverse and dynamic driving environments, often resulting in lower detection accuracy when confronted with complex scenes. As a consequence, these methods frequently fall short of meeting the stringent demands for precise lane detection required in autonomous driving applications.

The structure of this paper is as follows. Section 2 details the related methods and their limitations. Section 3 introduces the design of the lane detection network model. Section 4 introduces the design of the cross-convolutional hybrid attention module. Section 5 discusses the training and testing of the model using the road lane detection dataset. Section 6 concludes the paper.

## Related work

With the advancement of hardware and the development of deep learning, lane line detection technology has also progressed significantly, greatly enhancing the ability to extract and recognize lane lines. Pan et al.^16^ proposed the Spatial Convolutional Neural Network (SCNN) detection model, which promotes the fusion of information across different regions in the feature map and captures global features, allowing for better detection of the global structural information of lane lines. Gomaa et al.^17^ introduced a lightweight residual structure combined with channel attention mechanisms to adaptively enhance multi-scale feature representation while suppressing irrelevant background interference. Neven et al.^18^ introduced an end-to-end detection algorithm based on instance segmentation. The end-to-end learning model does not require predefined parameters and directly extracts lane line information from the image. Haris et al.^19,20^ proposed the AK-CNN network model, which reduces computational load by designing asymmetric kernel convolutions and improves detection speed with a three-branch convolutional structure based on asymmetric kernels. Additionally, a multi-scale spatial convolution structure was designed to extract features at various scales, reducing the loss of information in adjacent local regions and thereby enhancing detection accuracy. Pang et al.^21^ proposed the Fast-Hybrid Branch Network (Fast-HBNet), a network that incorporates a hierarchical feature learning module capable of learning lane line features at different scales, channels, and spatial levels, improving the network’s generalization ability. Wu et al.^22^ introduced the DBSCAN clustering algorithm for instance segmentation, which can detect lane lines in varying quantities. Gomaa et al.^23^ proposed a feature extraction strategy integrating morphological operations with motion-based clustering, demonstrating robustness in handling dynamically changing scenes. Mei et al.^24^ proposed a multi-task deep convolutional network aimed at identifying the geometric attributes of lanes, in combination with recurrent neural networks for lane line detection. Liu et al.^25^ introduced a top-down lane detection framework. CondLaneNet solves the issue of distinguishing lane instances by incorporating conditional lane detection strategies. Zheng et al.^26^ proposed the ROIGather module, which enhances feature representation by collecting global context and introduces Line IoU loss to treat lane lines as a unified entity, improving localization accuracy. Despite their advancements, these methods still face certain limitations in handling dynamic and complex scenarios, especially when addressing global context information and long-range dependencies.

The attention mechanism can dynamically focus on important regions within an image, which aids in more accurate extraction of lane line information. Hou et al.^27^ proposed a self-attention distillation method, where attention maps are extracted at a certain stage of the network model’s training for self-distillation. This approach complements segmentation supervision learning by using the attention map from the previous layer as the distillation target, enabling layer-by-layer attention distillation, which effectively extracts lane lines. Alotaibi et al.^28,29^ developed a semi-automatic framework that integrates modified detection architectures with background subtraction and clustering techniques to minimize manual annotation efforts. Guo et al.^30^ designed a lightweight convolutional structure and utilized a dual-attention mechanism to enhance detection accuracy and improve runtime performance. Zhao et al.^31^ introduced the Spatial Recurrent Feature-Shift Aggregator (SPRESA) module, which aggregates spatial information and reduces computational load by combining spatial attention mechanisms. Yang et al.^32^ addressed the issue of slow clustering and segmentation speed by employing a self-attention mechanism, improving lane segmentation accuracy. Deng et al.^33^ proposed a dual-branch feature adaptive fusion method for lane line detection, which uses channel attention and self-attention mechanisms to guide feature fusion and adaptively adjusts the fusion process. Umirzakova et al.^34^ proposed the MIRA-CAP framework, which employs memory-integrated retrieval-augmented methods for efficient scene understanding in images and videos, demonstrating the effectiveness of cross-modal feature fusion in dynamic environments. However, due to the complex and dynamic nature of road environments, along with the variety of interference factors, the aforementioned methods rely on pre-trained lane line data and are effective only for specific interference factors, making them unsuitable for adapting to the ever-changing driving environment.

To enhance our algorithm’s focus on slender lane regions in images while reducing the influence of non-lane features on detection results, we propose a lane detection model based on the Cross-Convolutional Hybrid Attention Mechanism (CCHA-Net). We combine channel attention and spatial attention with cross convolution to smooth lane features, maintain the continuity of lane markings in the feature map, and improve detection accuracy in complex and dynamic environments.

## Design of lane line detection network model

**Fig. 1:**
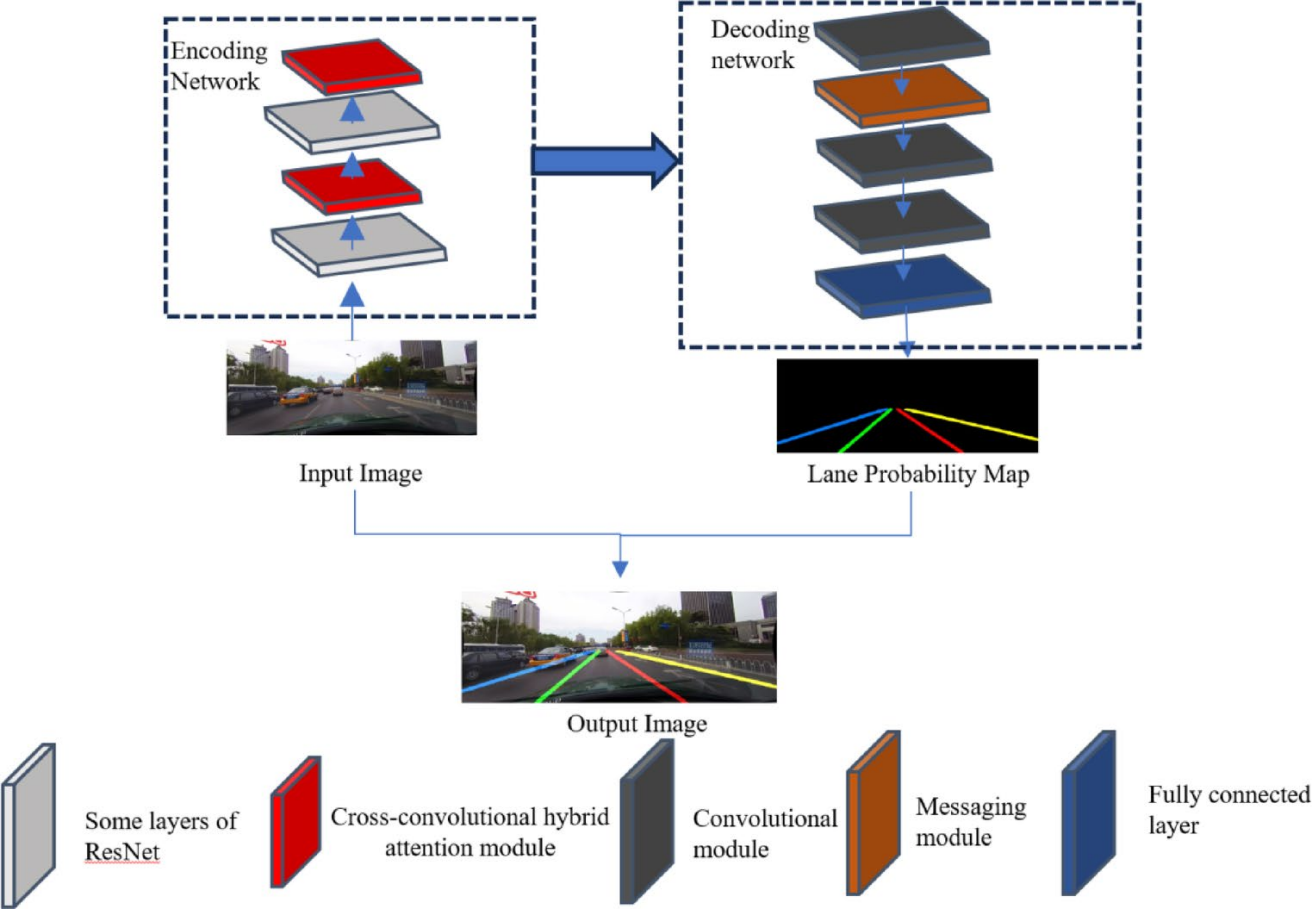
Overall detection network model.

The overall structural framework of the lane line detection network model proposed in this paper is shown in Fig. 1.

The model consists of an encoding network, a decoding network, partial layers of ResNet, a cross-convolutional hybrid attention module, convolutional modules, a message-passing module^16^, and a fully connected layer. The designs of the encoding network and decoding network are outlined as follows.

### Encoder network design

During feature extraction from images, the model’s accuracy improves as the network depth increases, reaching its peak at a certain depth. However, if the network continues to deepen, the model’s accuracy drastically drops. This issue arises due to the vanishing and exploding gradient problems that occur with deeper networks. ResNet mitigates this issue by introducing residual modules, allowing the output of a layer to skip one or more intermediate layers and connect directly to later layers, enabling the propagation of information without excessive gradient loss.

**Fig. 2:**
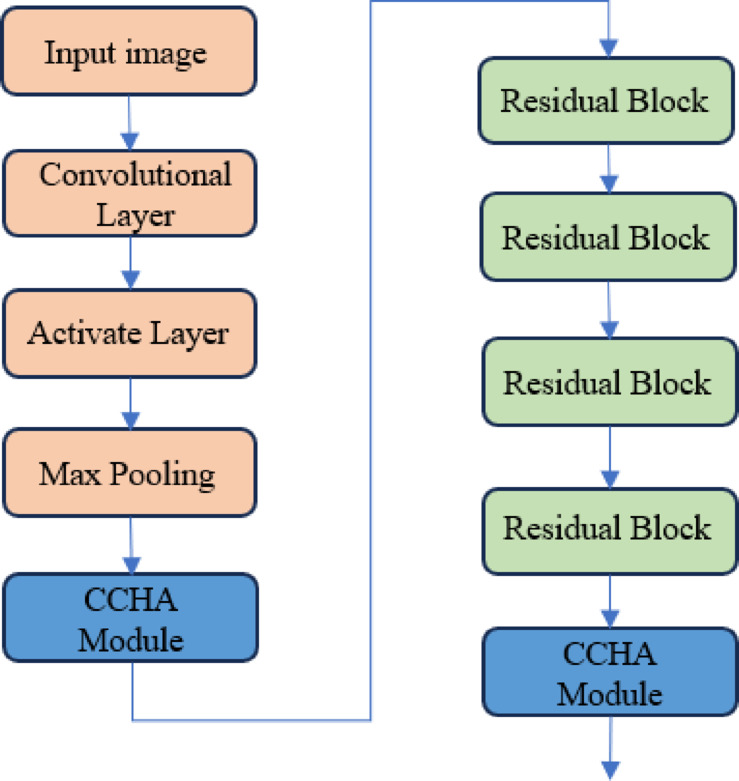
Encoding network.

The encoder of the CCHA-Net model, based on ResNet18’s partial layers, embeds the cross-convolutional hybrid attention mechanism into both shallow and deep feature extraction layers. This enhances the model’s ability to learn spatial and channel-wise features, assisting the model in capturing contextual information from different regions and scales. This embedding improves the model’s understanding and recognition of image content. The encoder network structure is illustrated in Fig. 2.

### Decoder network design

The decoding network primarily extracts lane line features from the encoder and decodes them for output. The CCHA-Net decoder consists of three convolutional modules, one message-passing module, and one fully connected layer. The decoder’s tasks include feature extraction, feature space transformation, upsampling to the original image size, and ultimately segmentation and existence prediction.

**Fig. 3:**
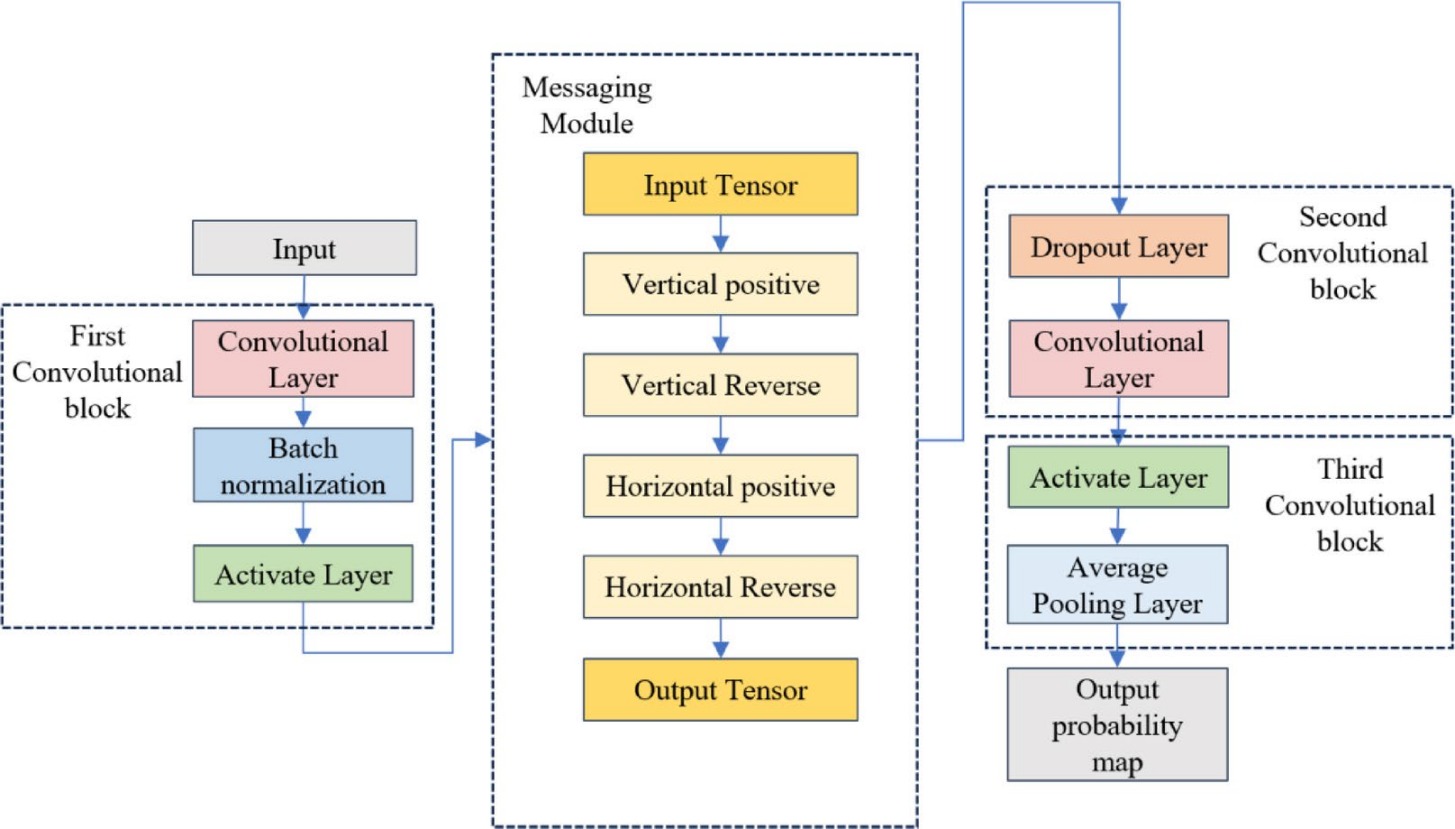
Decoding network.

The decoder network structure is shown in Fig. 3. First, lane line features extracted by the encoder pass through the first convolution block. This block extends the receptive field without increasing the number of parameters, capturing more contextual information. A batch normalization layer is introduced in the first convolution block to stabilize the learning process and speed up training. The activation layer uses a ReLU function to introduce non-linearity into the decoding network. The features then pass into the message-passing module, where a series of convolutional layers enhance the exchange of spatial domain information.

The message-passing module strengthens the model’s ability to capture long-range spatial dependencies in lane lines by propagating contextual information in multiple directions. This is achieved by dual-branch multi-scale convolutions that fuse cross-spatial domain features, with vertical (up-down) and horizontal (left-right) directions transmitting local features pixel by pixel. The vertical and horizontal convolutions use 1 × 5 and 5 × 1 kernels, respectively, and apply weight-sharing and bias-free designs to reduce parameters. When designing the convolution kernels for each convolution step, this paper uses a set of kernel parameters to perform convolutions across different regions or multiple channels, instead of learning separate parameters for each local area. This design allows the same convolution kernel to repeatedly extract features from the entire feature map, significantly reducing the total amount of parameters. During batch normalization, this paper uses a bias-free design by omitting the additional bias parameters in convolutional layers. The normalization layer partially compensates for the role of the bias, so removing it does not noticeably affect performance, while reducing the number of parameters and helping to prevent overfitting.

After the message-passing module, the second convolution block is introduced, further refining the features and reducing model overfitting. To map intermediate feature maps to the final segmentation prediction, the third convolution block includes a softmax activation function and an average pooling layer, assigning a class probability distribution to each pixel and reducing the spatial resolution of the feature map. Finally, a fully connected layer converts the feature map sequence into existence predictions, outputting values between 0 and 1, indicating the probability of lane line presence.

## Design of the Cross-Convolutional hybrid attention model

The human visual system has an attention mechanism that allows rapid scanning of the surrounding environment and focuses on specific key regions through internal signal processing. This mechanism helps humans filter out irrelevant background information and focus on valuable target information, improving the efficiency of visual data processing.

In deep learning, simulating this attention mechanism allows a model to quickly recognize and concentrate on the most important parts of the input data after training, enabling more efficient task processing and improved performance in handling complex visual data.

### Network architecture

**Fig. 4:**
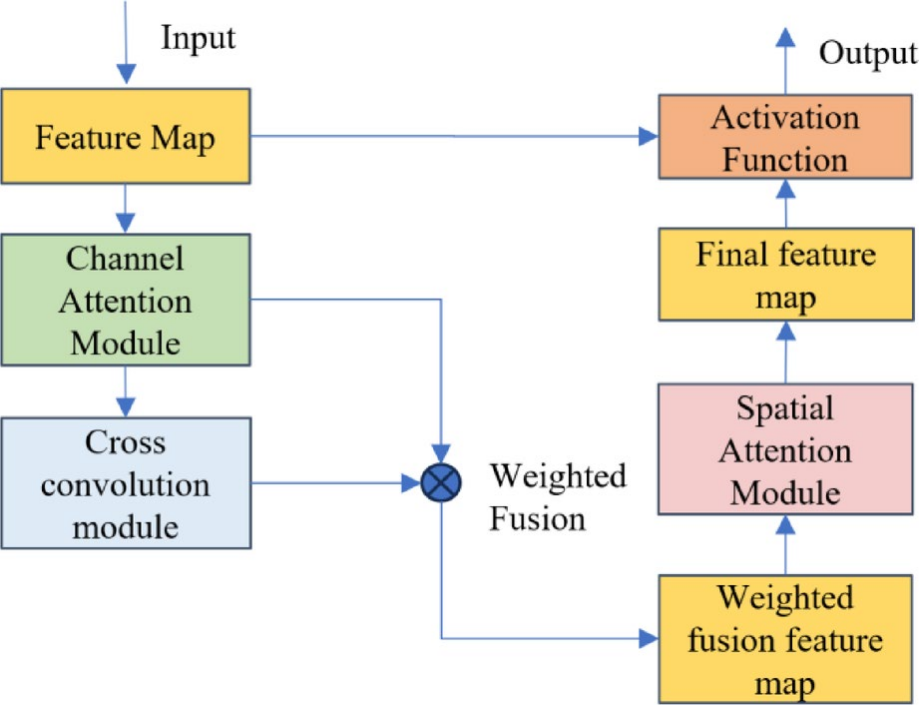
Cross-convolution hybrid attention module.

Lane detection tasks face challenges such as lighting variations (e.g., glare, shadows), lane wear, and occlusions, which can negatively affect detection performance. To address these issues and improve detection accuracy, we designed the cross-convolutional hybrid attention module, which helps the model identify and focus on lane line areas in the input data, thereby enhancing the model’s ability to understand and learn lane line information. The structure of the cross-convolutional hybrid attention model is shown in Fig. 4.

#### Establishment of channel attention module network model

**Fig. 5:**
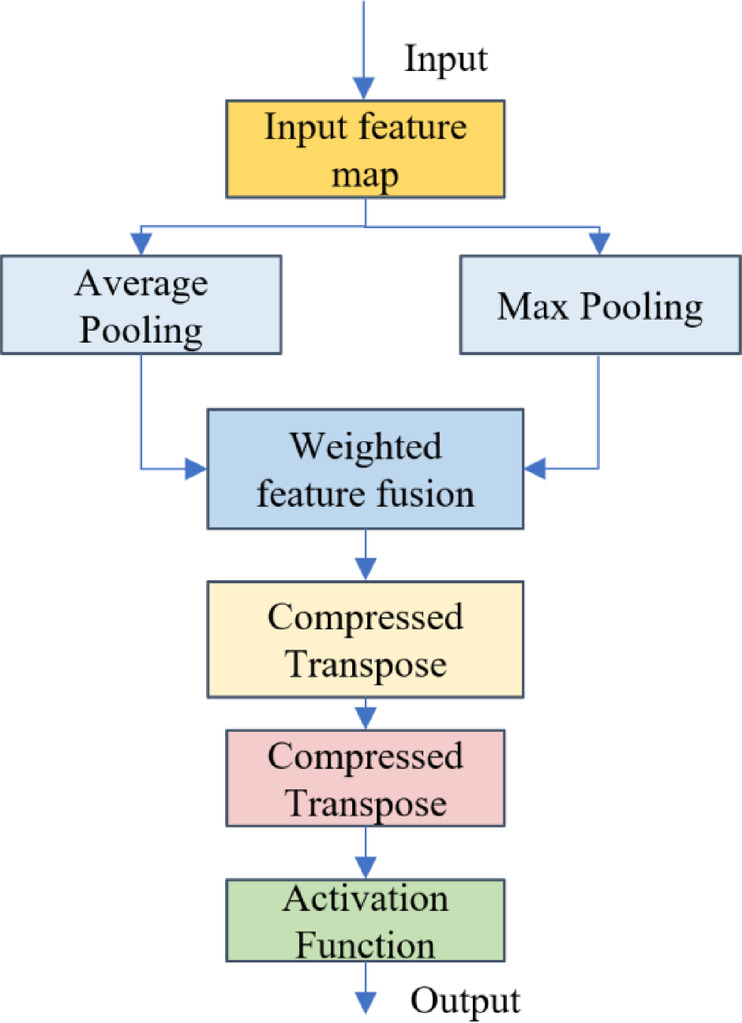
Channel attention module.

The network structure of the channel attention module is shown in Fig. 5. It models the importance of the channel dimension through multimodal statistical feature extraction and dynamic weight allocation mechanisms. The input feature map undergoes parallel global average pooling and global maximum pooling operations, which generate statistical features representing the average response strength F avg​ and the saliency peak F max​ between channels. To combine the advantages of these two statistical modalities, adaptive weight parameters α and β (initial value 0.5, trainable for optimization) are designed, and the fused feature F add​ is obtained through weighted summation. The calculation formula for the weighted fused feature is given by Eq. (1):

1 $$F{\text{ }}add=0.5*\left( {F{\text{ }}avg+F{\text{ }}max} \right)+\alpha *F{\text{ }}avg+\beta *F{\text{ }}max$$

This fusion strategy adaptively adjusts the global distribution and local saliency weights of channel features. To further enhance channel discriminability, this paper apply a 1D convolutional layer to the fused feature, compressing its dimension and activating it to generate the channel attention weight map. Finally, feature calibration is performed through channel-wise multiplication. This design, through dual-modal feature complementarity and dynamic weight learning, significantly improves the model’s sensitivity to key channel features, thereby enhancing robustness in complex scenarios.

The weighted fused feature map is compressed and transposed, then passed through a 1D convolutional layer to obtain a weight vector that represents the importance of each channel. The model applies the Sigmoid activation function to obtain the final channel attention weight. This final channel attention weight is then multiplied element-wise with the input feature map to produce the weighted feature map, map1.

The channel attention module enhances the network’s focus on the channel dimension of the feature map, allowing the model to adaptively adjust based on different channel features and improve its sensitivity to various features.

#### Establishment of Cross-Convolution module network model

**Fig. 6:**
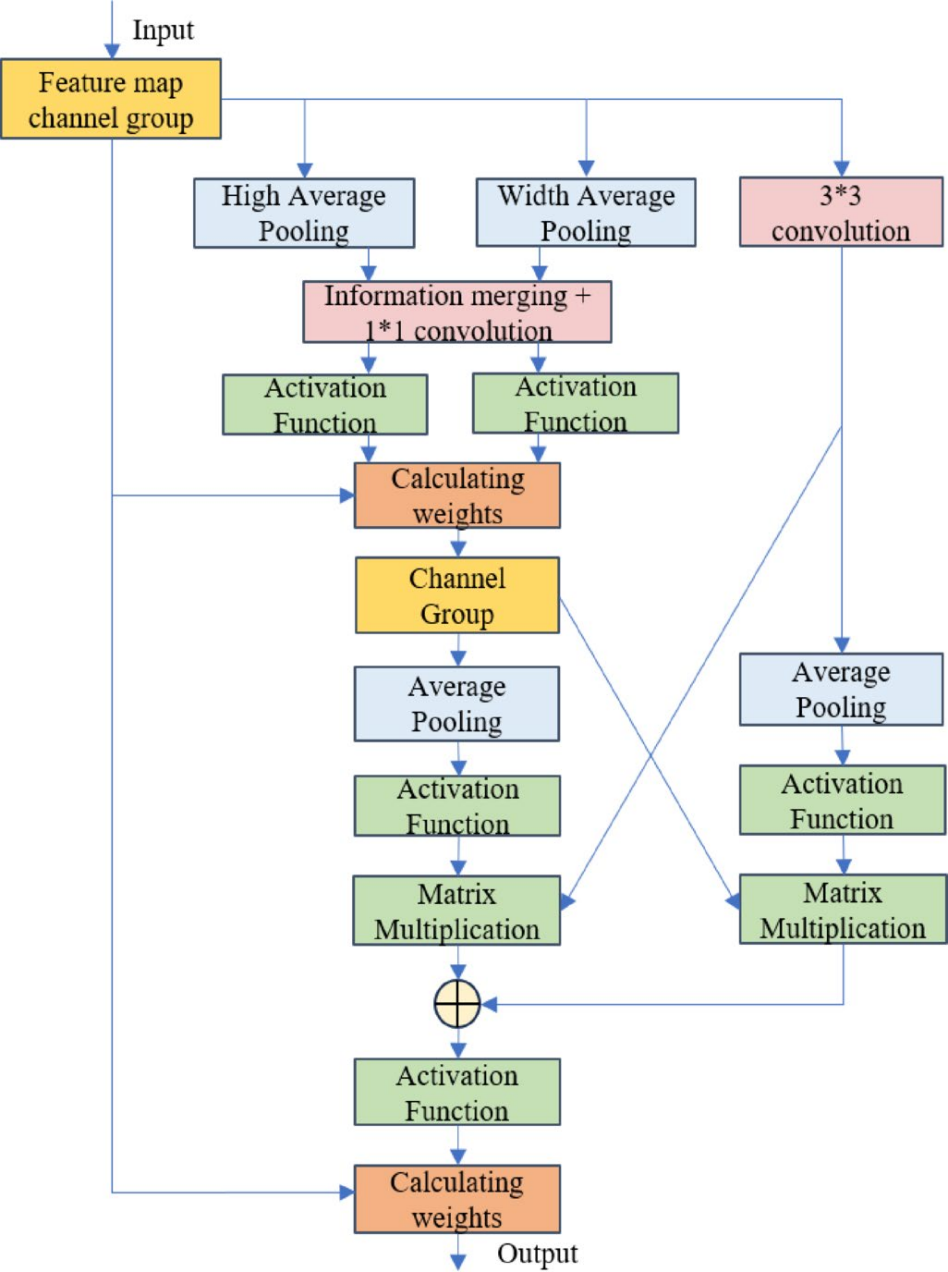
Cross convolution module.

The network structure of the cross-convolution module is shown in Fig. 6. Its objective is to enhance the continuity of lane features through hierarchical feature interaction and to integrate multi-scale spatial context information. The module first divides the weighted feature map output from the channel attention module into 32 feature groups along the channel dimension, enabling fine-grained processing of local features. For each feature group, average pooling is applied in the height and width directions to aggregate spatial statistical information, generating two compressed feature representations. These two feature maps are then concatenated along the channel dimension and fused through a 1 × 1 convolutional layer for multi-axis context information integration. The fused feature map is dynamically calibrated through element-wise multiplication with the original grouped features to generate a new feature map, Feature1, with group normalization introduced to stabilize the training process. This hybrid design effectively decouples the learning of channel and spatial features while maintaining computational efficiency, thereby strengthening the model’s ability to capture elongated lane structures.

On the other hand, the module further enhances the discriminability of channel features through multi-branch feature interaction and dynamic attention fusion mechanisms. For each feature group, the original feature map is processed by a 3 × 3 convolutional layer to extract local spatial details, generating fine-grained features, Feature2. Meanwhile, the context fusion feature, Feature1, generated in the previous step, undergoes adaptive average pooling to compress its spatial dimension and is standardized to generate global statistical weights. Both are normalized using the Softmax function, and matrix multiplication is performed to capture cross-branch feature correlations, generating a spatial-channel joint attention weight map. After reshaping, this weight map is element-wise multiplied with the original feature map and activated through a Sigmoid function to produce the refined feature map, map2. The final channel attention feature map is obtained by weighted summation of map1 and map2 using the defined parameter θ, as shown in Eq. (2):

2 $$channel{\text{ }}attention{\text{ }}map=map1*\theta +map2*\left( {1 - \theta } \right)$$

where *θ*∈[0,1] is optimized via backpropagation to balance the contributions of local details and global context.

The cross-convolution module smooths and stabilizes the weighted feature map output from the channel module, helping the network focus on the elongated regions of the lane lines in the feature map, suppressing irrelevant information and enhancing the feature expression capabilities.

#### Establishment of Spatial attention module network model

**Fig. 7:**
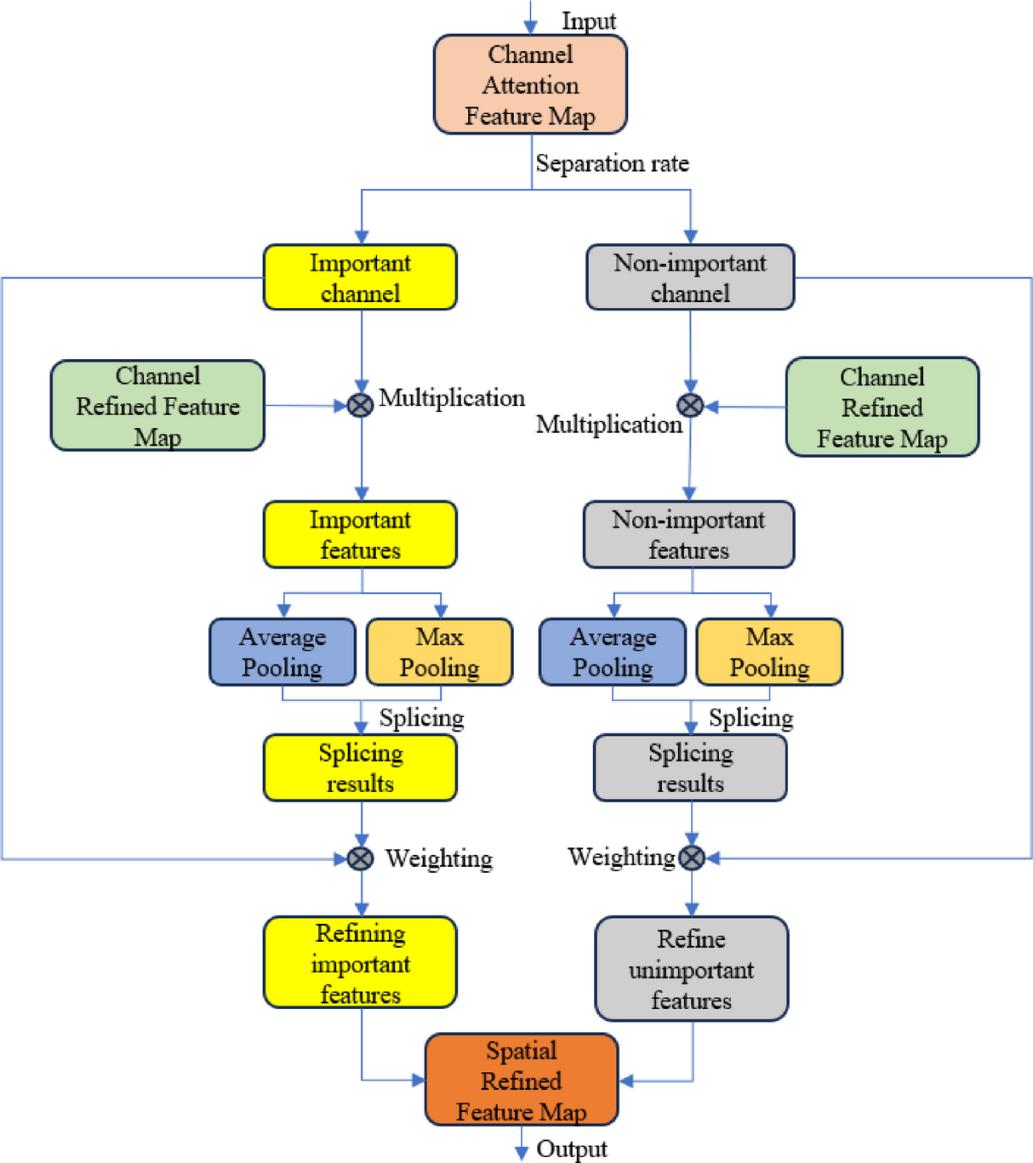
Spatial Attention Module.

The network structure of the spatial attention module is shown in Fig. 7. It adopts a dynamic channel selection mechanism and a multi-modal feature complementarity strategy, aiming to enhance the model’s ability to focus on spatial key regions. This module first dynamically classifies important and non-important channels based on the response strength distribution of the channel attention feature map, using an adaptive threshold separation algorithm. It generates binary masks to extract the key region features from the refined channel feature map.

To fully exploit the complementarity of these two feature types, a dual-modal statistical aggregation strategy is employed. One strategy enhances key features by applying global average pooling to capture spatial consistency patterns, while the other suppresses background noise by performing global maximum pooling on non-important features, reducing redundant responses.

The aggregated features are concatenated along the channel dimension and undergo a nonlinear transformation via a composite convolution-batch normalization-ReLU (Conv-BN-ReLU) unit, generating a spatial attention weight map. Finally, residual connections are used to merge the original features with the weighted features.

Through dynamic channel selection and multi-granularity feature complementarity, the model’s ability to perceive lane line edges and topological structures is significantly improved, especially exhibiting strong robustness in complex scenarios such as occlusions and uneven lighting conditions.

### Loss function

The loss function consists of two parts. One part is the segmentation loss, which represents the difference between the predicted segmentation and the ground truth segmentation labels. It is calculated using the cross-entropy loss function, as shown in Eq. (3)^35^:

3 $${\mathcal{L}_{{\text{seg}}}}= - \frac{1}{N}\sum\limits_{{i=1}}^{N} {\sum\limits_{{c=1}}^{C} {{w_c}} } \cdot {y_{i,c}}\log ({p_{i,c}})$$

where *N* is the total number of pixels, *C* is the number of classes (lane line class + background),${w_c}$ represents the class weight, which is inversely proportional to the class frequency,${y_{i,c}}$ is the true label for pixel i, and ${p_{i,c}}$ is the predicted probability that pixel *i* belongs to class *c*.

The other part is the existence loss, which represents the difference between the predicted existence and the ground truth existence labels. It is calculated using the binary cross-entropy loss function, as shown in Eq. (4)^35^:

4 $${\mathcal{L}_{{\text{exist}}}}= - \frac{1}{B}\sum\limits_{{b=1}}^{B} {\left[ {{y_b}\log ({{\hat {y}}_b})+(1 - {y_b})\log (1 - {{\hat {y}}_b})} \right]}$$

where *B* is the batch size, ${y_b}$∈{0,1} represents the lane line existence label for the b-th image, and ${\hat {y}_b}$ is the predicted probability of the lane line’s existence.

The total loss is the weighted sum of the segmentation loss and the existence loss.

## Experimental validation

The processor used for experimental validation is an 18 vCPU AMD EPYC 9754 128-Core Processor, with an RTX 4090 GPU and 24 GB of VRAM, running on Ubuntu 20.04. The model is implemented using the PyTorch deep learning framework, and the TensorRT inference framework is utilized to accelerate the neural network’s inference speed.

### Datasets

This paper uses the CULane and TuSimple datasets to train and test the model. The CULane dataset is a large-scale, diverse, and challenging dataset that includes various types of roadways such as urban, suburban, and highway roads. It also contains nine different scenarios: normal, shadow, curve, highlight, crowded, arrow, wireless, night, and intersection. The dataset allows for the annotation of up to four lane lines per image, enabling the evaluation of the model’s performance in detecting lane lines across different complex scenarios. The TuSimple dataset, on the other hand, only includes lane line data from U.S. highways under moderate weather conditions during the daytime at various times. The image resolutions, total number of frames, and the corresponding splits for the training, validation, and testing sets for both datasets are shown in Table 1.

**Table 1:** Lane line dataset.

Dataset	Total Frames	Training set	Validation set	Test Set	Resolution
CULane	133,325	88,880	9675	34,680	1640*590
TuSimple	6408	3268	358	2782	2782*720

To standardize and augment the input data, the proposed method employs a custom preprocessing pipeline built on OpenCV and PyTorch. This pipeline sequentially applies a series of transformations implemented as custom Python classes. Initially, the method resizes images to a fixed target size or performs random resizing by sampling width and height from predefined intervals (minW, maxW, minH, and maxH) to simulate scale variations. Next, the method applies random rotations, controlled by a parameter theta, to mimic different viewpoints and enhance robustness. Then, the method normalizes the images using mean and standard deviation values derived from the dataset, ensuring a consistent input distribution. Finally, the method converts the processed images into tensors and scales their values appropriately for model training. Preliminary experiments determine key parameter values—including target dimensions, rotation angle range, and normalization statistics—ensuring the preservation of critical lane features while introducing sufficient variability to improve model generalization.

### Evaluation metrics

To facilitate comparison with other lane detection algorithms, the official evaluation standard of the dataset is adopted. The CULane dataset considers lane markings as narrow curves with a width of 30 pixels. The dataset computes the Intersection over Union (IoU) between the predicted lane markings and the ground truth to evaluate the accuracy of the prediction. If the IoU is greater than 0.5, the lane marking is considered correctly predicted and classified as a true positive (TP); if it is less than 0.5, it is deemed incorrect and classified as a false positive (FP); lane markings that are not detected are considered false negatives (FN). Since there are no lane markings at intersections, FP values are used as the evaluation metric, with smaller FP values indicating better detection performance. For all other scenarios, the harmonic mean of precision and recall, known as F1 score, is used as the final evaluation metric. The formulas for precision, recall, and F1 score are given in Eqs. (5)-(7)^16^:

5 $${P_{precision}}={\text{ }}{N_{TP}}/\left( {{N_{TP}}+{N_{FP}}} \right)$$

6 $${P_{recall}}={\text{ }}{N_{TP}}/\left( {{N_{TP}}+{N_{FN}}} \right)$$

7 $${P_{F1}}={\text{ }}2{P_{precision}}{P_{recall}}/\left( {{P_{precision}}+{P_{recall}}} \right)$$

In the equations, *P*_*precision​*_ represents precision; *P*_*recall​*_ represents recall; *P*_*F1*​_ represents the harmonic mean F1 score; *N*_*TP*_ ​represents the number of true positives; *N*_*FP*_​ represents the number of false positives; and *N*_*FN*_​ represents the number of false negatives.

The TuSimple dataset has three evaluation metrics, namely False Positive Rate (FPR), False Negative Rate (FNR), and Accuracy. Their respective calculation formulas are presented in Eqs. (8)-(10)^36^:

8 $${P_{FPR}}={N_{FP}}/{N_{pred}}$$

9 $${P_{FNR}}={N_{FN}}/{N_{gt}}$$

10 $$Paccuracy=\frac{{\sum\limits_{i} {{C_i}} }}{{\sum\limits_{i}^{{}} {{S_i}} }}$$

In the equations, *P*_*FPR*_ denotes the False Positive Rate; *N*_*FP*_ represents the number of lane lines incorrectly identified as lane lines from non-lane regions; *P*_*FNR*_ refers to the False Negative Rate; *N*_*FN*_ indicates the number of actual lane lines that were not detected; *P*_*accuracy*_ denotes the Accuracy; *N*_*pred*_ refers to the total number of predicted lane lines; *N*_*gt*_ represents the total number of ground truth lane lines; *C*_*i*_ is the number of correctly predicted lane points in the i-th image; *S*_*i*_ is the number of actual lane points in the i-th image.

### Comparative experiments

The results of SCNN, UFLD, LaneATT, FastDraw, CLRNet, CondLaneNet, and the proposed CCHA-Net are compared in various scenarios on the CULane dataset, as shown in Table 2. The proposed algorithm achieves the best detection performance in normal, shadow, curve, and arrow scenarios, with an overall F1 score of 80.2%. Segmentation-based methods, such as SCNN, face challenges in maintaining lane smoothness because they do not treat the lane lines as a cohesive entity for prediction. CondLaneNet generates lane proposals from a single starting point, which may lead to the omission of certain lane instances. In contrast, the proposed method outputs continuous and smooth lane line predictions even in complex scenarios, demonstrating its strong capability in capturing global context and its superior detection accuracy.

**Table 2:** Detection results of CULane dataset.

Scenario	SCNN^16^	UFLD^37^	Lane-ATT^38^	Fast-Draw^39^	CLR-Net^26^	CondLane-Net^25^	CCHA-Net
Normal /%	90.6	90.7	91.2	85.9	93.3	92.8	**93.6**
Shadow /%	66.9	69.3	68.0	69.9	79.6	80.0	**80.3**
Curve /%	64.4	69.5	63.8	65.2	71.5	72.4	**73.3**
Highlight/%	58.5	59.5	65.8	57.0	**73.7**	70.7	70.4
Crowd/%	69.7	70.2	72.7	63.6	**78.3**	75.7	76.0
Arrow/%	84.1	85.7	87.8	79.4	**90.2**	89.3	**90.2**
Noline/%	43.4	44.4	49.1	40.6	**53.1**	52.3	51.2
Night/%	66.1	66.7	68.6	57.8	**75.1**	72.2	72.8
Cross/per	1990	2037	**1020**	7013	1321	1364	1341
Entirety	71.6	72.3	75.1	–	79.5	78.1	**80.2**

**Fig. 8:**
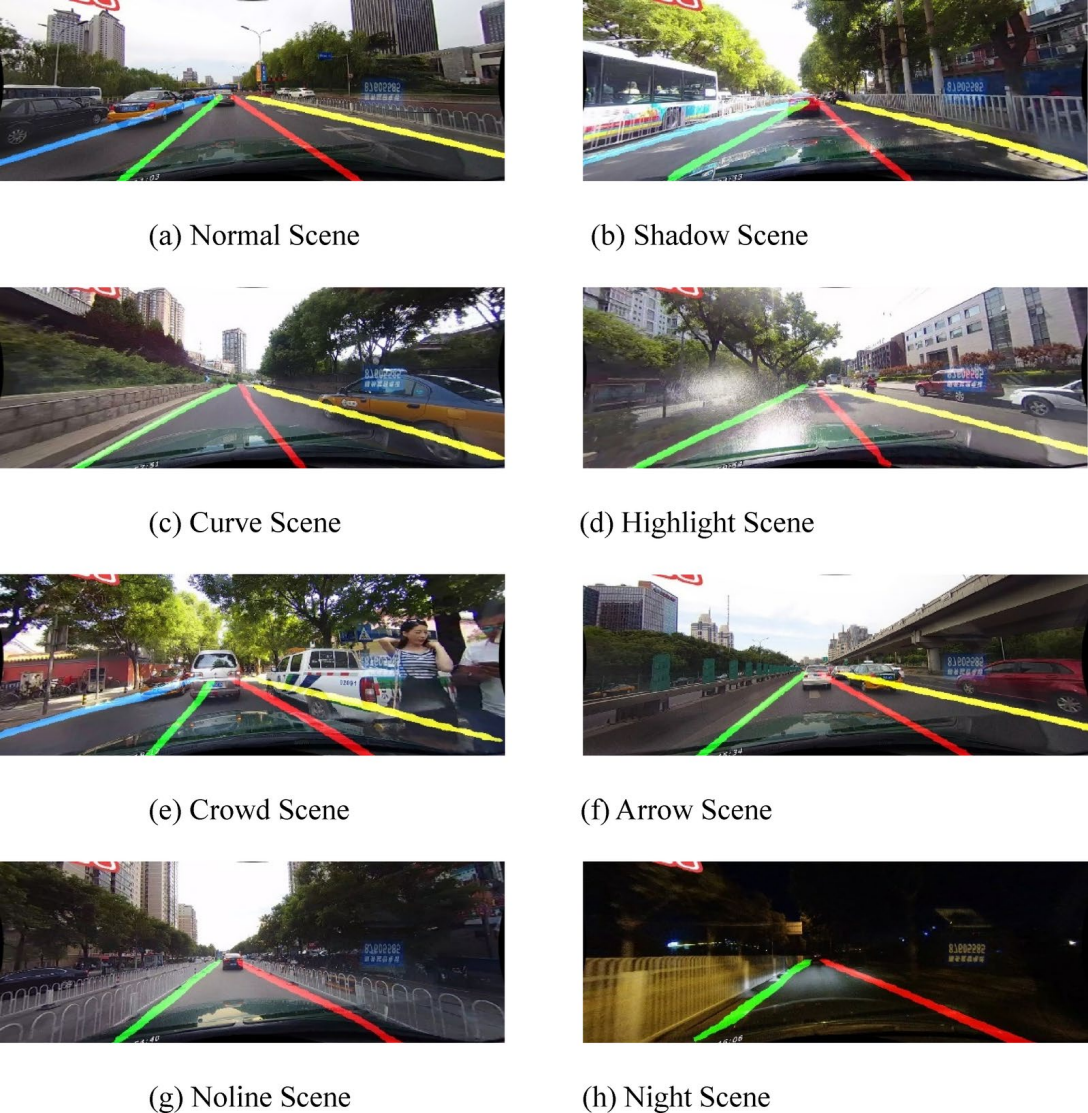
Accurate real lane line detection results of different scenarios using CCHA module smooth features.

The proposed algorithm can accurately identify lane line information across different scenarios. Figure 8 illustrates the detection results of the proposed algorithm in various scenarios, as shown in the figure.

A comparison of the SCNN, SAD, LaneNet, UFLD, E2Enet, PolyLaneNet, LSTR, and the proposed CCHA-Net algorithms was conducted on the TuSimple dataset, with results presented in Table 3. Since the TuSimple dataset consists of high-quality highway lane line images under favorable conditions, all detection algorithms achieved relatively good detection results. The proposed algorithm achieved the highest accuracy and the lowest false positive rate, with a significant reduction in the false negative rate. This indicates a lower likelihood of misclassifying background areas as lane lines, which is of significant importance for lane keeping in autonomous driving.

**Table 3:** TuSimple dataset detection results.

Method	Accuracy/ %	False Positive Rate/ %	False negative rate /%
E2Enet	96.0	3.2	4.3
LaneNet	96.4	7.8	2.4
LSTR	96.2	2.9	3.4
PolyLaneNet	93.4	9.4	9.3
SAD	96.6	6.0	2.1
SCNN	96.5	6.2	**1.8**
UFLD	95.9	18.9	3.8
CCHA-Net	**96.8**	**2.1**	2.8

This paper evaluates the practicality of CCHA-Net in resource-constrained environments by analyzing its computational efficiency; the FLOPs and parameters of CCHA-Net are measured using the thop library and compared with existing methods. The results are shown in Table 4.

**Table 4:** Hardware efficiency comparison on CULane dataset.

Method	FLOPs /G	Params (M)	F1/%
SCNN	20.3	10.5	71.6
UFLD	1.2	0.8	72.3
LaneATT	6.7	3.9	75.1
CLRNet	90.2	26.8	79.5
CCHA-Net	5.5	3.6	80.2

Table 4 compares the hardware efficiency and performance of various lane detection algorithms on the CULane dataset. UFLD, with 1.2G FLOPs and 0.8 M parameters, emerges as the model with the lowest computational complexity. Its design pursues extreme computational efficiency by adopting a lightweight network architecture and row classification strategy, which maintain low FLOPs and parameter counts. However, UFLD achieves an F1 score of 72.3%, significantly lower than that of the proposed CCHA-Net at 80.2%. The lightweight backbone and row classification strategy reduce complexity at the expense of global feature modeling capacity, limiting UFLD’s ability to handle scenarios with occlusions or abrupt illumination changes. In contrast, CCHA-Net employs a cross-convolution mixed attention mechanism to dynamically fuse channel and spatial features, enhancing lane continuity representation, and integrates a multi-scale message passing module to optimize the detection of curved or discontinuous lanes. CCHA-Net achieves a significant accuracy breakthrough—improving the F1 score by 7.9%—with 5.5G FLOPs and 3.6 M parameters, and reduces error rates by 34.8–50% under extreme conditions such as nighttime and high-glare, fully validating its advantage in balancing efficiency and robustness in complex dynamic environments.

### Ablation experiments

To validate the improvement effect of the proposed Cross-Convolution Hybrid Attention Module and Cross-Convolution Module on lane line detection, experiments were conducted by removing these modules from the model and training with the same parameters on the TuSimple dataset. The experimental results are shown in Table 5. The complete model with the Cross-Convolution Hybrid Attention Module achieved better results.

**Table 5:** Network model module ablation comparison results.

Model	Accuracy/ %	False Positive Rate/ %	False Negative Rate /%
Remove the cross-convolution mixed attention module	90.3	10.7	9.8
Only remove the cross convolution module	95.1	6.6	3.5
Complete Model	96.8	2.1	2.8

**Fig. 9:**
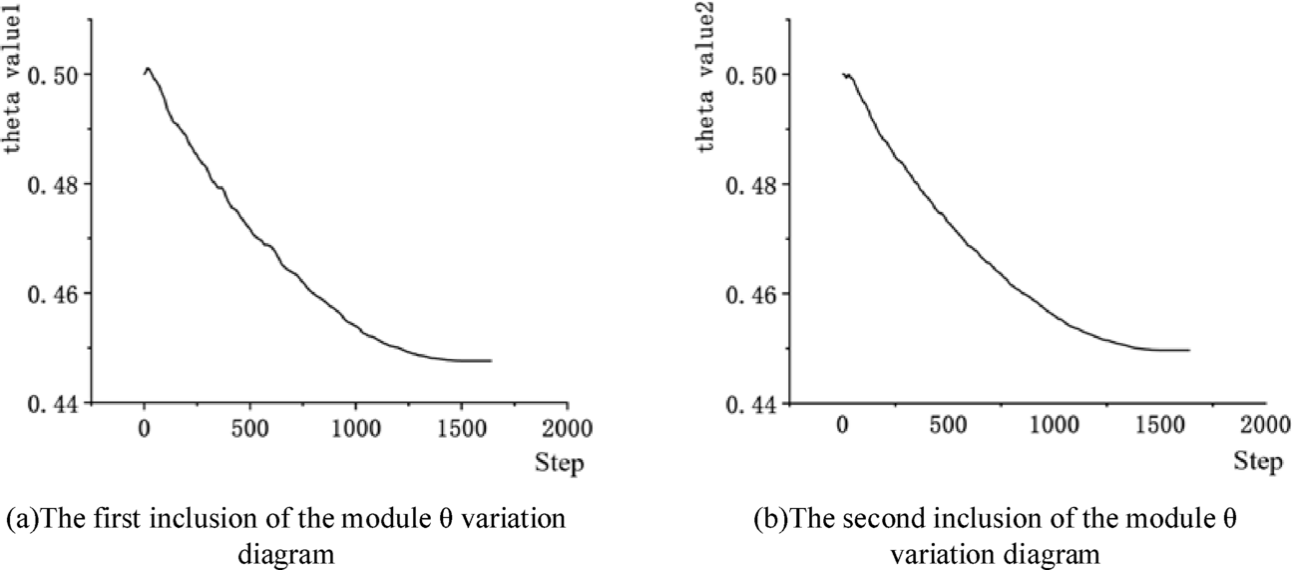
theta change curve.

The values of the variable parameter θ during the training process were monitored in TensorBoard. The change curves of θ in the two instances of the Cross-Convolution Hybrid Attention Module are shown in Fig. 9. As observed from the figure, the values of θ in both instances decrease overall as the training steps increase. According to Eq. (2), the weights of the original channel attention feature map continuously decrease, while the weights of the channel attention feature map, after being smoothed by the Cross-Convolution Module, continue to increase. This indicates that the Cross-Convolution Module plays an active role in lane line feature extraction.

When predicting the existence of lane lines, the network processes multi-layer fused image features through a fully connected layer to generate four numerical outputs corresponding to four potential lane lines. The Sigmoid activation function maps these values into the 0–1 range, representing the existence probability of each lane line.

**Fig. 10:**
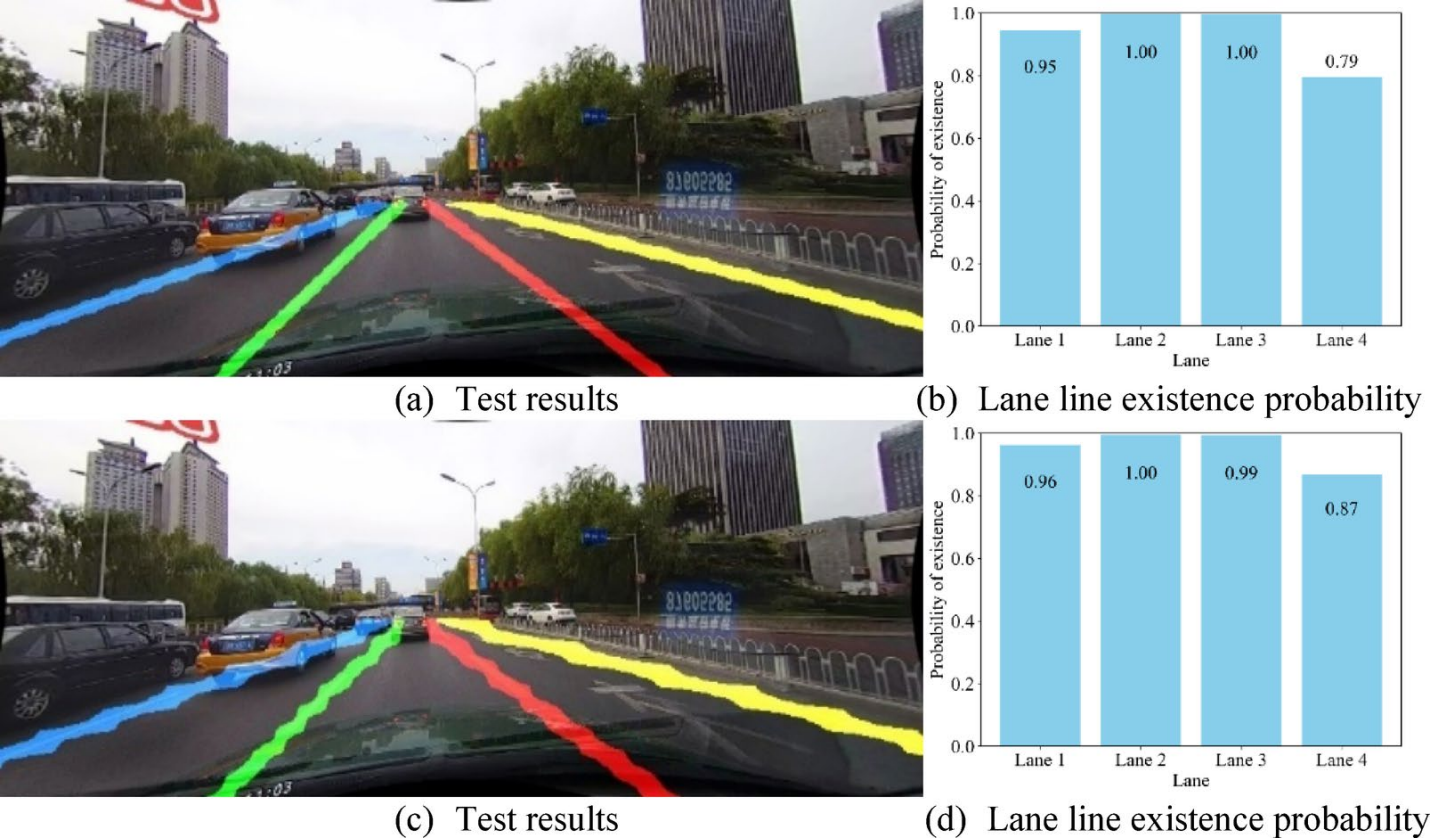
Comparative visualization of normal scene detection results.

**Fig. 11:**
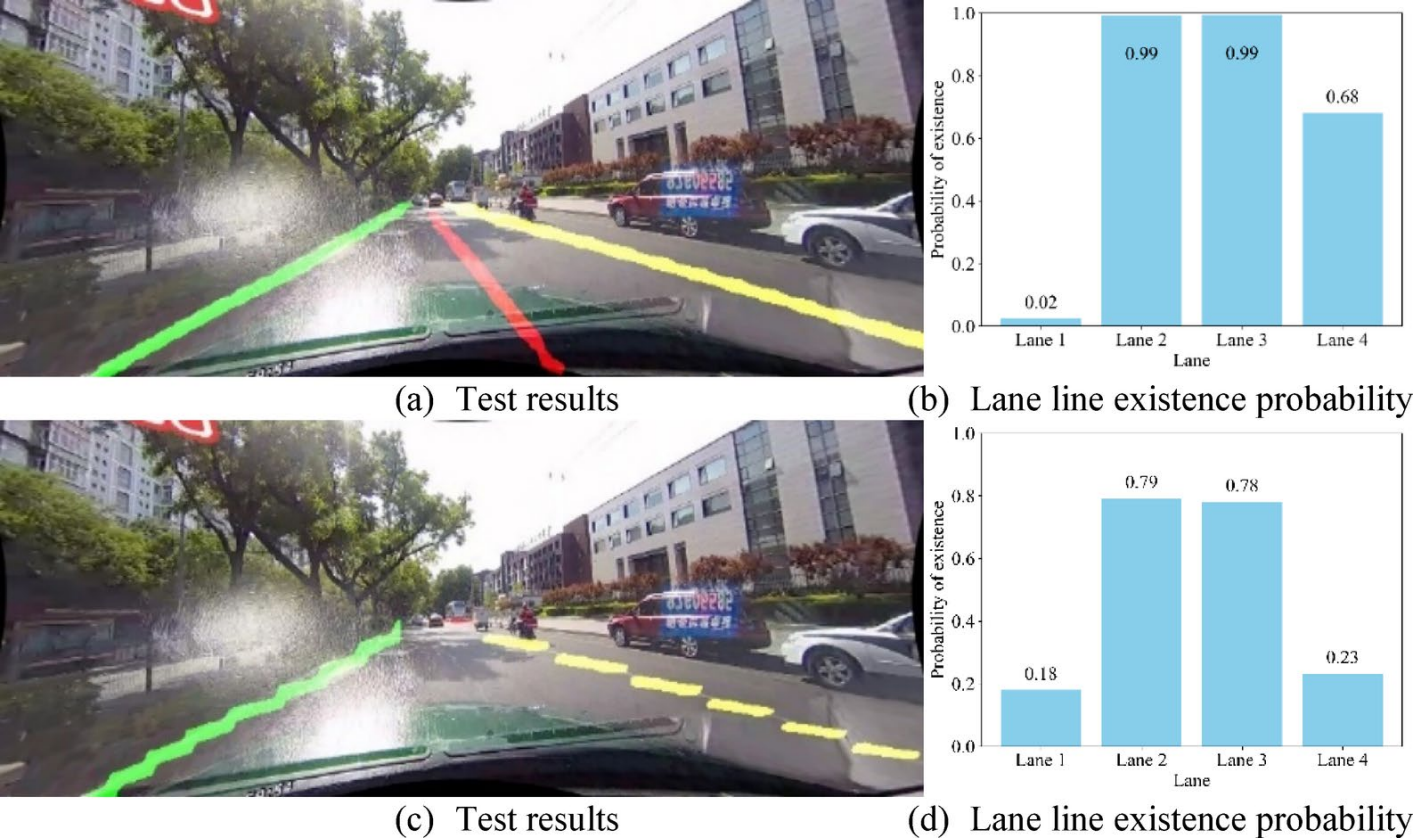
Comparative visualization of hignlight scene detection results.

To visually demonstrate the effectiveness of the proposed Cross-Convolutional Hybrid Attention (CCHA) module, this study compares detection results from network models with and without this component. The evaluation employs both normal scene and highlight lane line images, extracting existence probabilities for each lane line during detection. The analysis compiles the existence probabilities of all lane lines in the images into bar charts. Figures 10 and 11 present comparative visualizations of detection results between the baseline and CCHA-enhanced models.

Figures 10(a) and 10(b) present the detection results and lane line existence probability maps of the CCHA-enhanced model for normal scene images, while Figs. 10(c) and 10(d) display the corresponding outputs of the baseline model without CCHA. Similarly, Figs. 11(a) and 11(b) illustrate the detection results and probability maps of the CCHA-equipped model for highlihght scene, with Figs. 11(c) and 11(d) showing the baseline model’s performance under identical conditions.

The comparative results demonstrate that the CCHA-enhanced model achieves more precise lane line detection with superior continuity. For actual existing lane lines, predictions closer to 1 indicate higher detection accuracy, while probabilities approaching 0 for non-existent lane lines reflect more reliable negative detection. Both scenarios show that the CCHA-integrated model delivers more accurate predictions for both present and absent lane lines compared to the baseline.

To quantify performance, we evaluated 10 images per scenario by calculating an error metric: for each lane line, the error equals 1 − p (where p represents the predicted probability of an existing lane line) or p (for non-existent lane lines). The image-level error rate is the average sum of all lane line errors within the image. Table 6 statistically summarizes the average detection error rates across different scenarios.

**Table 6:** Average detection error rate in different scene.

Scene	Error rate with CCHA module	Error rate without CCHA module
Normal	0.07	0.12
Shadow	0.1	0.15
Curve	0.12	0.18
Highlight	0.09	0.13
Crowd	0.07	0.1
Arrow	0.08	0.1
Noline	0.15	0.23
Night	0.09	0.18

Table 6 demonstrates that the CCHA module significantly reduced the detection error rate across all eight scenarios, achieving an average reduction of 0.06. Notably, in the Night scene, the error rate decreased from 0.18 to 0.09 (a 50% reduction), while the Noline scene exhibited a reduction from 0.23 to 0.15 (a 34.8% decrease), underscoring the module’s robust adaptability to complex environments.

The CCHA module achieved greater error reduction in scenarios involving illumination interference (e.g., Night, Highlight) or structural complexity (e.g., Curve, Noline) compared to simpler scenes. This performance gap highlights the module’s superior capability in noise suppression and feature enhancement, particularly under challenging conditions.

### Real-world testing

**Fig. 12:**
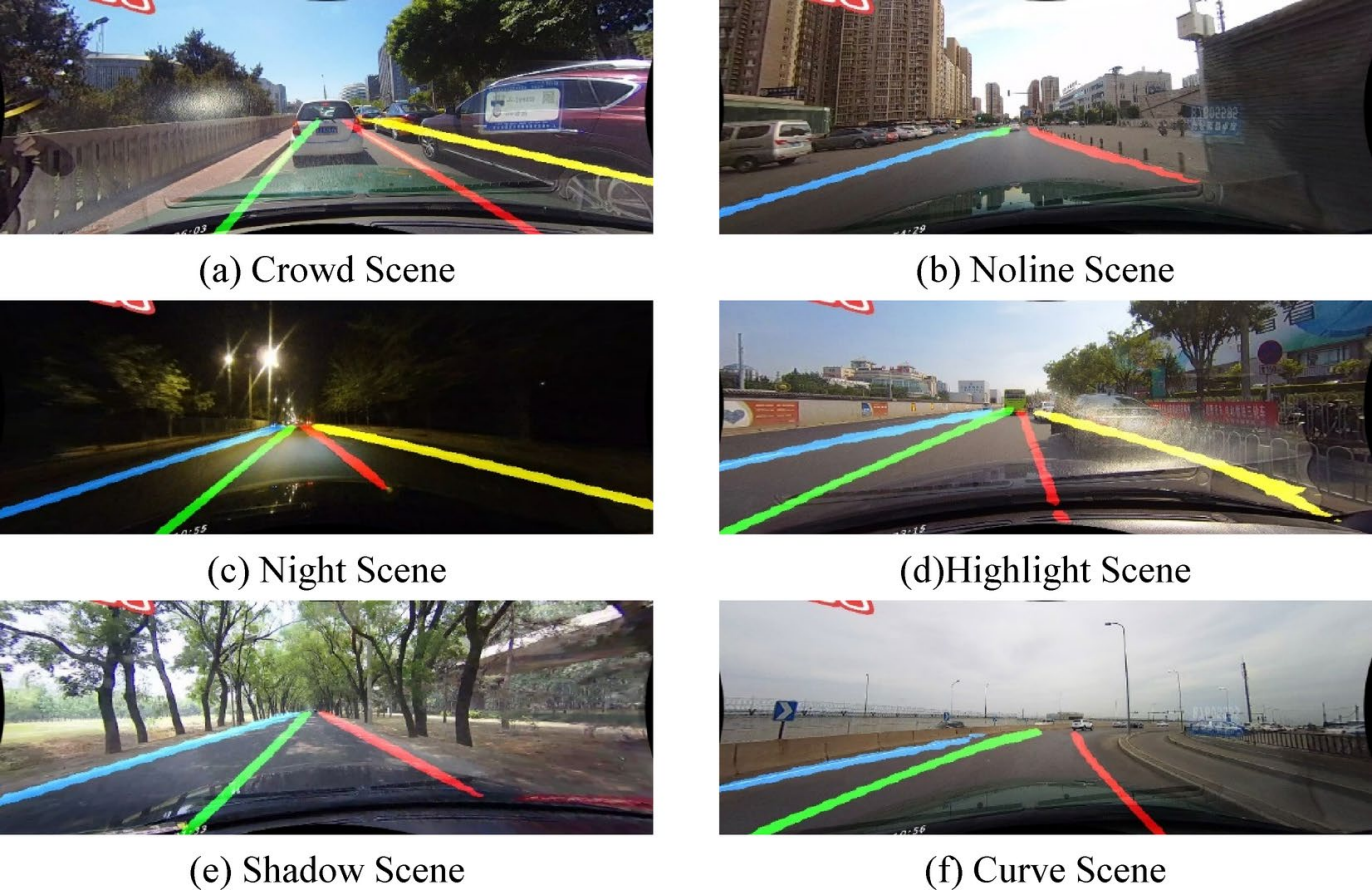
Accurate real lane line detection results of different scenarios using CCHA module smooth features.

This paper collected actual road images and tested the CCHA-Net network model trained on the CULane dataset. The test results are shown in Fig. 12. The results demonstrate that the proposed algorithm can accurately identify lane markings in complex real-world road environments, indicating that the algorithm exhibits strong robustness. This paper built the network model using the CULane and TuSimple datasets for training. These two datasets do not include rainy snowy and foggy scenarios so the model does not learn the lane line image features in those conditions. As a result the model fails to accurately identify lane lines in such environments.

## Conclusion

Lane detection is a critical component of autonomous driving, and ensuring the accuracy of lane detection in complex and dynamic environments is a key factor in evaluating lane detection technologies. This paper proposes a lane detection algorithm, CCHA-Net, based on a Cross Convolution Hybrid Attention Mechanism. The proposed algorithm does not require predefining the number of lanes and is capable of detecting lane markings in various complex traffic scenarios. To enhance feature extraction capabilities, a Cross Convolution Hybrid Attention module is designed and integrated into the lightweight backbone network ResNet18. During the decoding process, a message-passing module is incorporated to improve the accuracy of lane detection. Experimental results show that the F1 score on the CULane dataset reaches 80.2%, while the accuracy on the TuSimple dataset is 96.8%, with a false positive rate of 2.1% and a false negative rate of 2.8%. We validated the algorithm in real-world road scenarios to demonstrate its practical applicability for lane detection in actual road environments.

## Data Availability

The datasets analysed during the current study are available in the CULane repository, https://xingangpan.github.io/projects/CULane.html. The datasets analysed during the current study are available in the TUSimple repository, https://github.com/TuSimple/tusimple-benchmark/issues/3.
